# Characterisation of Lamp2-deficient rats for potential new animal model of Danon disease

**DOI:** 10.1038/s41598-018-24351-w

**Published:** 2018-05-02

**Authors:** Shuoyi Ma, Miao Zhang, Shuai Zhang, Jing Wang, Xia Zhou, Guanya Guo, Lu Wang, Min Wang, Zhengwu Peng, Changcun Guo, Xiaohong Zheng, Xinmin Zhou, Jingbo Wang, Ying Han

**Affiliations:** 1 State Key Laboratory of Cancer Biology, National Clinical Research Centre for Digestive Diseases and Xijing Hospital of Digestive Diseases, Fourth Military Medical University, Xi’an, China; 2 Division of Ultrasonography, Xijing Hospital, Fourth Military Medical University, Xi’an, China; 3 Division of Psychiatry, Xijing Hospital, Fourth Military Medical University, Xi’an, China

## Abstract

Danon disease (DD) is caused by the absence or malfunction of lysosomal-associated membrane protein 2 (LAMP2). Although *Lamp2*-deficient mice and DD patients have similar characteristics, these mice have clear limitations and are clinically inconsistent. The aim of our paper is to outline the characteristics of *Lamp2*-deficient rats and to contrast this model with currently available DD mouse models. The baseline levels of some serum enzymes were elevated in *Lamp2*^y/−^ rats along with hypercholesterolemia and hyperglycaemia at 8 weeks. Echocardiography showed that IVSd (1.500 ± 0.071 vs. 2.200 ± 1.147, P < 0.01) and LVPWd (1.575 ± 0.063 vs. 1.850 ± 0.029, P < 0.01) were significantly increased, and GCS (−13.20 ± 0.4814 vs. −6.954 ± 0.665) and GRS (21.42 ± 1.807 vs. 7.788 ± 1.140) were sharply decreased. Meanwhile, substantial myocyte disruption, hypertrophic muscle fibres, interstitial fibrosis and microvascular hyperplasia could be observed in the heart tissue. Lamp2^y/−^ rats also displayed abnormal behaviours in the open field and fear conditioning tests. Notably, Lamp2^y/−^ rats manifested other system dysfunctions, such as retinopathy, chronic kidney injury and sterility. Based on these results, *Lamp2*-deficient rats exhibited greater similarity to DD patients in terms of onset and multisystem lesions than did mouse models, and these rats could be used as a valuable animal model for DD.

## Introduction

Danon disease (DD) is a rare monogenic X-linked disorder characterized by clinical traits of cardiomyopathy, myopathy and mental retardation^1^. While hypertrophic cardiomyopathy is the most frequent and life-threatening manifestation of the disorder in >85% male or >75% female patients^2^, not only multisystem phenotypic variability, such as neurological, gastrointestinal, respiratory, hepatic, ophthalmologic and psychiatric symptoms, but also considerable differences in the severity, age of onset and clinical outcomes between male and female patients have recently been described^3^. Due to the very low incidence of DD, comprehensive clinicopathology and natural history are difficult to obtain. Hence, well-characterized animal models for DD are essential to develop novel pathogenetic concepts and treatment strategies.

The molecular basis of DD derives from the absence or malfunction of lysosomal-associated membrane protein 2 (LAMP2), an abundant lysosomal membrane protein with an important role in immunity, macroautophagy and chaperone-mediated autophagy. The *Lamp2* gene is mapped to chromosome Xq24, and its mutations or microduplications could be pathogenic variations^4^. Histopathological features of *Lamp2*-deficient mice display the accumulation of unprocessed autophagosomes in a wide range of cell types and tissues, including skeletal and cardiac muscle, liver and platelets. Except for increased mortality and reduced size, this model also exhibits hypertrophic cardiomyopathy and intellectual dysfunction, as reported in human patients. *The Lamp*2-deficient mouse model of DD has been used in multiple studies to better characterize the disease. However, although these mice display many features similar to those observed in humans with DD, clear differences have also been reported. First, female homozygote mice are used as DD animal models, but patients are mostly males, and female homozygote patients remain asymptomatic or have non-typical symptoms in clinic^5–7^. Second, male homozygous mice do not have onset until middle age (6–10 months), while male patients often have early-age onset and die at adolescence^5,6^. Third, although the mouse model develops severely reduced cardiac contractility, the cardiac phenotype in humans seems more severe, and the cardiomyocyte vacuolation appears to be more pronounced compared with *Lamp2*-deficient mice^8,9^.

To clarify the DD phenotypic features, we generated *Lamp2*-deficient rats, which manifested striking intrahepatic cholestasis with liver function abnormalities^10^. Notably, these rats displayed similarity in severity, age of onset and clinical outcomes with human patients. Male hemizygous rats manifested reduced weight, and all died before postnatal day 80. In addition, these rats might suffer from male sterility and ophthalmologic disease besides myocardium and skeletal muscle lesions. Thus, *Lamp2*-deficient rats could be a more valuable animal model for DD than *Lamp2*-deficient mice. In this paper, we comprehensively investigated the phenotypic characteristics of *Lamp2*-deficient rats and the rats’ significance as a DD animal model.

## Results

### Early death and multisystem damage of *Lamp2*-deficient rats

In our previous study, *Lamp2*-deficient SD rats were created by TALEN Genome Editing Technology^10^. No *Lamp2*^−/−^ offspring was generated after monitoring for nearly 3 years. Therefore, we evaluated the effects of phenotypes of *Lamp2-*deficient rats using heterozygous *Lamp2*^+/−^ and hemizygous *Lamp2*^y/−^ rats. *Lamp2*^+/−^ showed no changes in weight, size or lifespan compared with wild-type rats, but *Lamp2*^y/−^ showed significantly reduced weight and size (Supplementary Fig. 1a) and shortened lifespan up to 80 days, which was consistent with patient deaths in adolescence. In addition, baseline levels of many serum enzymes were increased in *Lamp2*^y/−^ rats. At 8 weeks, troponin I, myoglobin and creatine kinase (CK) increased 18.53-fold, 3.82-fold and 1.41-fold, respectively. Alanine aminotransferase (ALT), aspartateaminotransferase (AST) and alkaline phosphatase (ALP) levels, all liver function indicators, were markedly higher. Meanwhile, *Lamp2*^y/−^ rats also displayed abnormalities of metabolic function with hypercholesterolemia (CHOL 5.21 ± 0.98 mmol/L) and hyperglycaemia (GLU 9.11 ± 1.57 mmol/L), but total protein (TP) and albumin (ALB) were intact (Table 1). Given that DD predominantly occurs in young males, we further investigated the potential for using hemizygote *Lamp2*^y/−^ rats as an animal model for DD.

**Table 1:** Biochemical characteristics of wild-type rats and Lamp2y/− rats at 8 weeks (n = 8/group).

Characteristics	Wild-type rats (n = 8)	*LAMP2*-deficient rats (n = 8)	Fold change	*p* value^a^
AST (IU/L)	198 ± 9.6	1411 ± 103.7	7.13	0.012^*^
CK (IU/L)	459 ± 14.7	646 ± 20.6	1.41	<0.0001^**^
CK-MB (IU/L)	2065 ± 302.1	1825 ± 226.1	0.88	0.543
LDH-1 (IU/L)	883 ± 100.1	850 ± 92.4	0.96	0.818
LDH (IU/L)	1635 ± 126.3	1527 ± 169.2	0.93	0.621
HBDH(IU/L)	933 ± 149.5	1048 ± 175.7	1.12	0.633
troponin I(ng/ml)	0.314 ± 0.06	5.820 ± 0.75	18.53	<0.0001^**^
myoglobin(ng/m)	17 ± 1.7	65 ± 7.5	3.82	0.012^*^
ALT (IU/L)	28 ± 5.1	93 ± 19.3	3.32	0.009^**^
TP (g/L)	68 ± 2.8	64 ± 3.1	0.94	0.301
ALB (g/L)	32 ± 1.0	29 ± 1.8	0.91	0.167
TBIL(mg/dL)	0.68 ± 0.33	0.73 ± 0.25	1.07	0.906
ALP (IU/L)	214 ± 37.9	476 ± 84.9	2.22	0.014^*^
TG(mmol/L)	0.93 ± 0.52	0.81 ± 0.56	0.87	0.877
GLU(mmol/L)	5.32 ± 2.14	9.11 ± 1.57	1.71	0.053
CHOL(mmol/L)	2.51 ± 0.69	5.21 ± 0.98	2.07	0.041^*^
BU(mmol/L)	5.1 ± 0.56	8.9 ± 2.26	1.74	0.149
CRE(μmol/L)	58 ± 5.3	42 ± 3.8	0.72	0.081
UA(μmol/L)	46 ± 5.6	70 ± 10.6	1.52	0.093

AST, aspartate aminotransferase; CK, creatine kinase; LDH, lactate dehydrogenase; CK-MB, MB isoenzyme of creatine kinase; HBDH, hydroxybutyrate dehydrogenase; ALT, alanine aminotransferase; TP, total protein; ALB, albumin; TBIL, total bilirubin; ALP, alkaline phosphatase; BU, blood urea; CRE, creatinine; UA, Uric acid. ^a^Student’s t-test for two groups. Mean ± SEM. *P < 0.05; **P < 0.01.

### *Lamp2*-deficient rats shows hypertrophic cardiomyopathy

**Figure 1:**
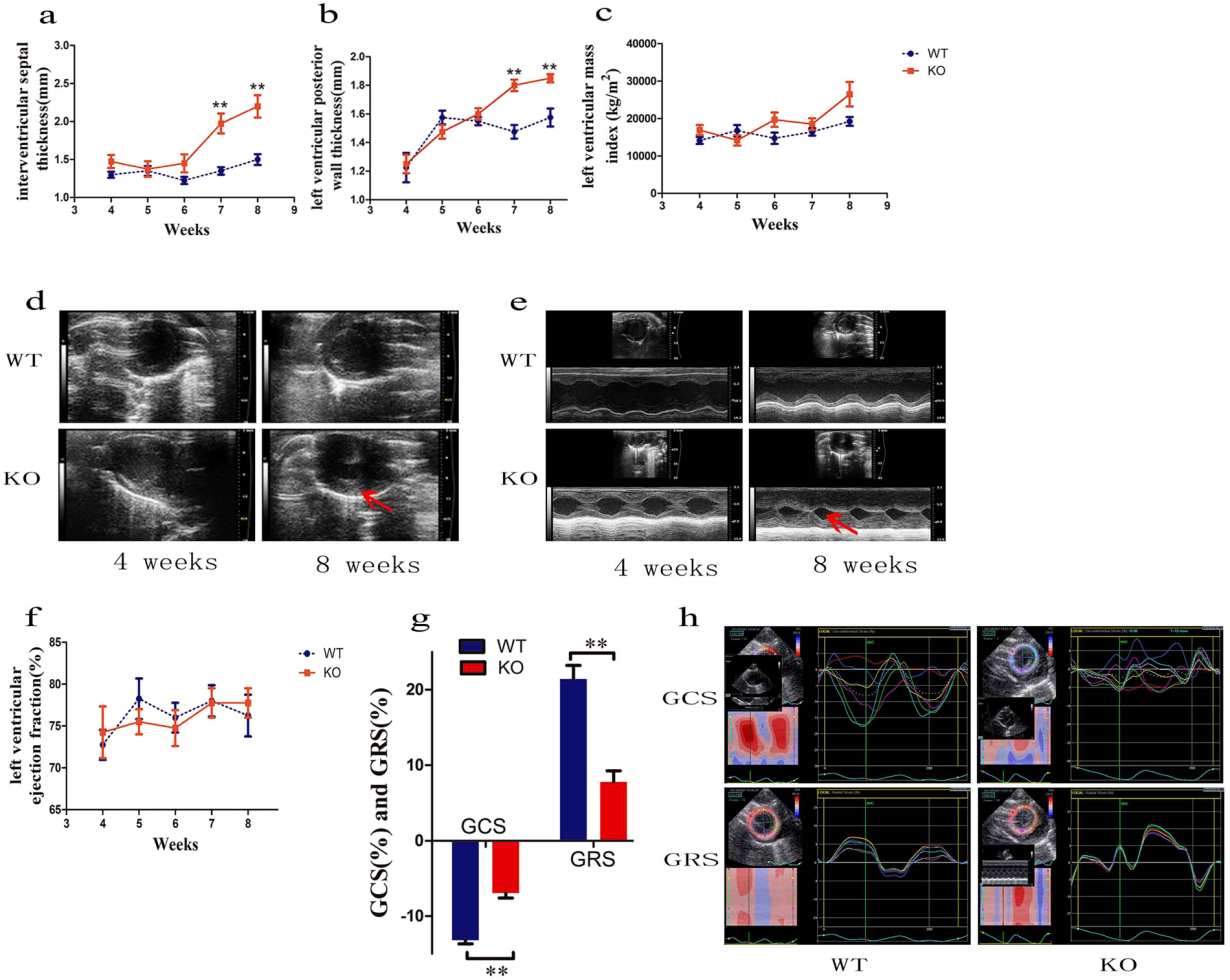
Echocardiographic parameters and representative echocardiographic images of *Lamp2*^y/−^ rats and wild-type rats. (**a**) IVSd; (**b**) LVPWd; (**c**) LVMI; (**d**) Echocardiographic image of left ventricular wall and dimensions; (**e**) Echocardiographic images of left ventricular systolic function; (**f**) LVEF; (**g**) GCS, GRS; (**h**) Echocardiographic images of GCS and GRS. Mean ± SEM, n = 10 rats per group. ^*^P < 0.05; ^**^P < 0.01. WT, wild-type male rats; KO, *Lamp2*^y/−^ rats.

As cardiomyopathy is usually the major defined characteristic of DD^11^, and *Lamp2*^y/−^ rats displayed damage to the myocardium with increased serum troponin I, myoglobin and CK, we first assessed the cardiac status of *Lamp2*^y/−^ rats. Echocardiography analysis showed that the baseline interventricular septum thickness in diastole (IVSd) increased with age in *Lamp2*^y/−^ rats, and a significant difference was observed at 7 weeks of age compared with wild-type rats (1.350 ± 0.050 vs. 1.975 ± 0.131, *P* < 0.01) (Fig. 1a and d). The baseline left ventricular posterior wall thickness in diastole (LVPWd) was also significantly higher at 7 weeks (1.475 ± 0.048 vs. 1.800 ± 0.041, *P* < 0.01) (Fig. 1b and d). There was no significant difference in left ventricular mass index (LVMI), but an elevated trend was found after 6 weeks in *Lamp2*^y/−^ rats (Fig. 1c). *Lamp2*^y/−^ rats exhibited not only thickened structure but also abnormal function. Although left ventricular ejection fraction (LVEF) (Fig. 1e and f) was comparable between *Lamp2*^y/−^ rats and wild-type rats, global circumferential strain (GCS) and global radial strain (GRS, Fig. 1g and h) were significantly reduced in *Lamp2*^y/−^ rats at 8 weeks.

**Figure 2:**
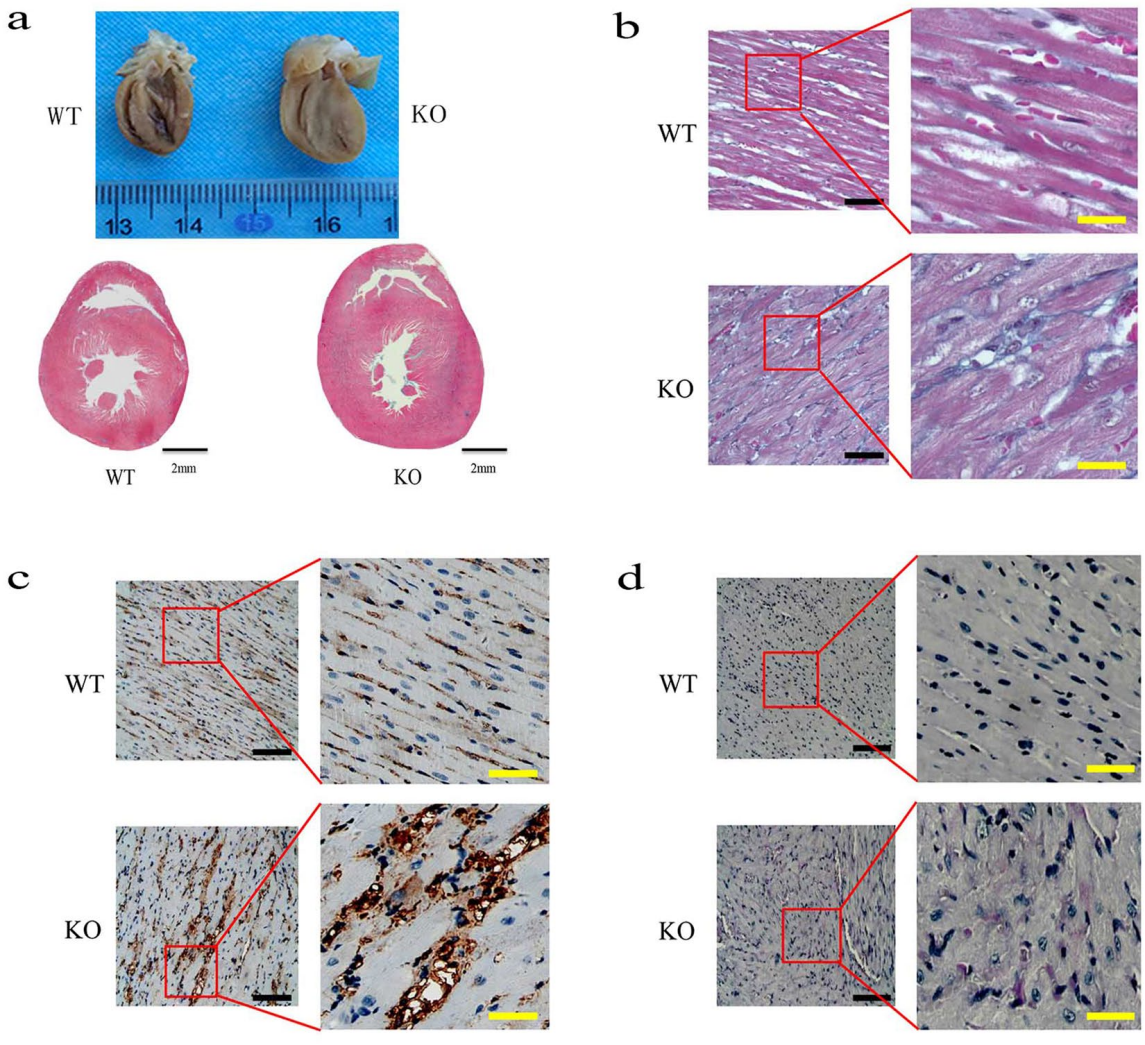
Histopathology of myocardium from *Lamp2*^y/−^ rats and wild-type rats (8 weeks). (**a**) Gross morphology of the heart; (**b**) Masson Trichrome staining; (**c**) Immunohistochemical reactivity for MAb B7, a marker for myocardium microvascular endothelial cells^32^; (**d**) Periodic Acid Schiff (PAS) staining. Bars in (**a**) correspond to 2 mm. The black bars correspond to 200 μm, and the yellow bars correspond to 2 mm in (**b**,**c** and **d**). WT, wild-type male rats; KO, *Lamp2*^y/−^ rats.

To investigate myocardium characteristics in *Lamp2*^y/−^ rats, we further explored the myocardium gross morphology and microscopic structure. Total heart weight in *Lamp2*^y/−^ rats was elevated compared with wild-type littermates (0.902 ± 0.082 vs. 1.340 ± 0.053, *P* < 0.001 Fig. S1b). The ratio of heart to body weight (0.004 ± 0.0004 vs. 0.012 ± 0.0006, *P* < 0.001, Fig. S1c) in *Lamp2*^y/−^ rats was much higher due to their lower body weight (Fig. S1b). Light microscopic analysis also demonstrated that heart size remarkably increased, cavity dimensions sharply shrank, and the ventricular wall thickened asymmetrically in *Lamp2*^y/−^ rats (Fig. 2a). Notably, the prominent microscopic structure of hypertrophic cardiomyopathy, substantial myocyte disruption, hypertrophic muscle fibres, increased interstitial collagen content and microvascular hyperplasia could be observed in the heart tissues of *Lamp2*^y/−^ rats (Fig. 2b). In addition, glycogen deposition was observed, indicating metabolic disturbance in myocardial cells (Fig. 2d).

### *Lamp2*-deficient rats have cognitive and locomotion dysfunction

**Figure 3:**
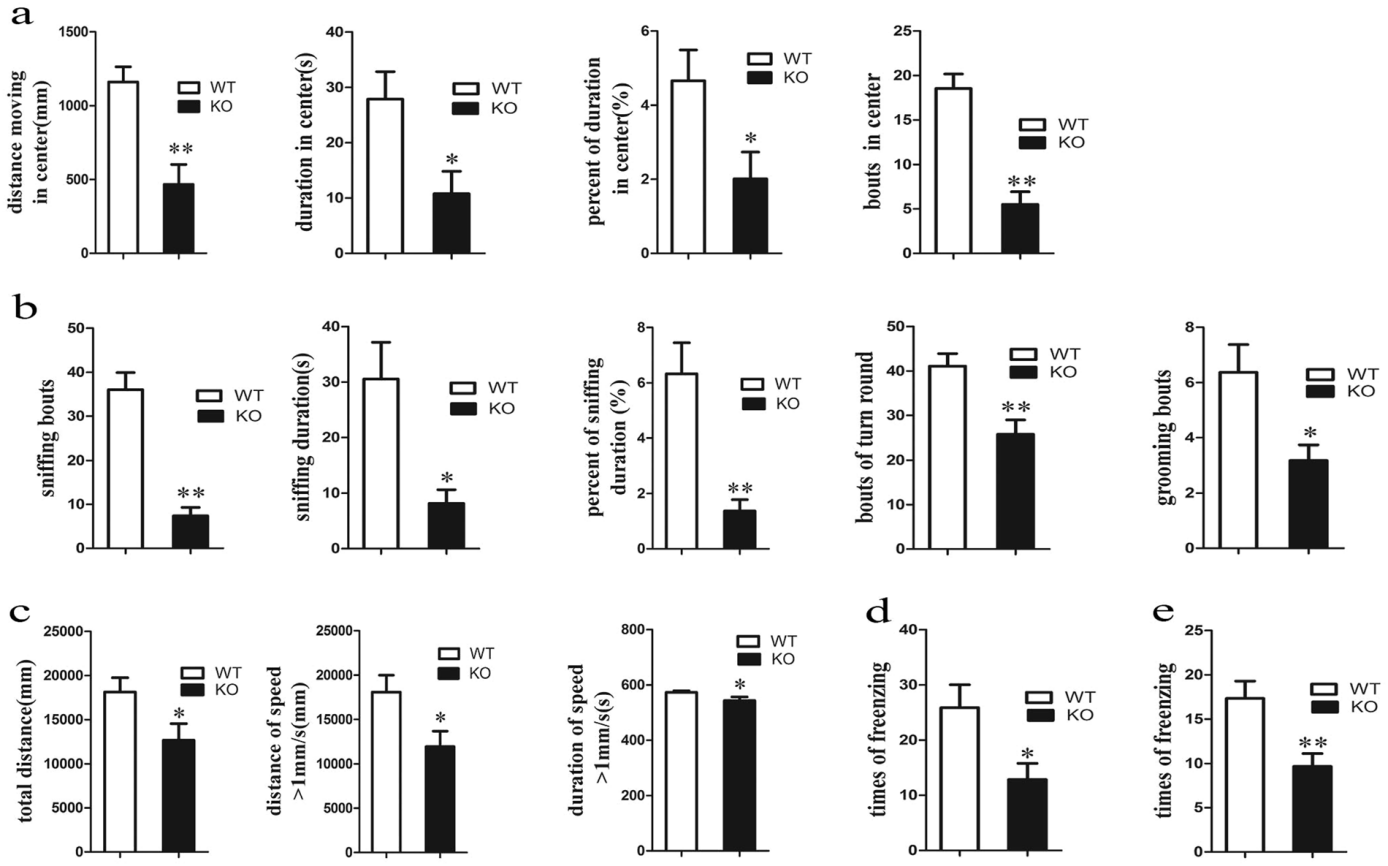
Behavioural tests including open field test and fear conditioning test in *Lamp2*^y/−^ rats and wild-type rats. (**a**) Distance, duration, percent of duration, and bouts in the central field; (**b**) Sniffing bouts, sniffing duration, percent of sniffing duration, bouts of turn round, and bouts of grooming in the field; (**c**) Total distance, distance of speed over 1 mm/s, duration of speed over 1 mm/s in the field; (**d**) Freezing times in contextual fear conditioning; (**e**) Freezing times in cued fear conditioning. Mean ± SEM, n = 10 per group. WT, wild-type male rats; KO, *Lamp2*^y/−^ rats.

Cognitive disorder is another prominent feature of DD; therefore, *Lamp2*^y/−^ rats were subjected to evaluation of exploration, locomotion, and memory abilities using the open field and fear conditioning tests. The open field test showed that distance, duration, percent of duration and bouts in the central area (Fig. S2a) were not significantly different compared with wild-type rats at 4 weeks, but they were significantly reduced at 8 weeks in *Lamp2*^y/−^ rats (Fig. 3a). Likewise, spontaneous activities in the field including sniffing bouts, duration and percent of duration, bouts of turn round and grooming (Figs 3b and S2b) were also decreased in *Lamp2*^y/−^ rats. Considering the severely reduced distance and duration in the central field as well as the frequency of spontaneous activities in the field, *Lamp2*^y/−^ rats indicated not only anxiety but lack of ability to explore in a novel environment. In addition, impairment of locomotion was detected due to significantly lower total distance and distance and time at a speed over 1 mm/s in the field (Fig. 3c).

Next, a fear conditioning study was performed to test short-term memory. No significant differences in freezing time between *Lamp2*^y/−^ and wild-type rats were observed (Fig. S2d and e). Nonetheless, there was a significant decrease in freezing times for both contextual and cued fear conditioning (Fig. 3d and e) in *Lamp2*^y/−^ rats. We speculated that freezing times were decreased markedly, while freezing time was unaltered due to the consequence of impaired motor performance. In conclusion, *Lamp2*^y/−^ rats showed reduced exploration, locomotion dysfunction and defective short-term memory.

**Figure 4:**
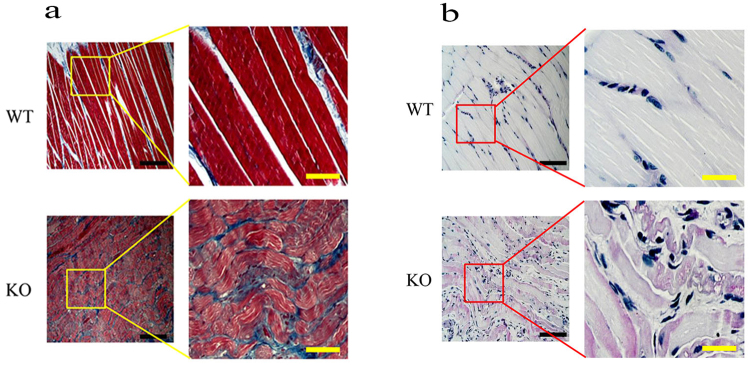
Histopathology of skeletal muscle from *Lamp2*^y/−^ rats and wild-type rats (8 weeks). (**a**) Masson Trichrome staining; (**b**) PAS staining. The back bars correspond to 200 μm, and the yellow bars correspond to 2 mm. WT, wild-type male rats; KO, *Lamp2*^y/−^ rats.

As disordered activation of the quadriceps muscle in DD is also well-known^12^, and impaired locomotion behaviours were found in *Lamp2*^y/−^ rats, we subsequently assessed the effect of *Lamp2* deficiency on skeletal muscles. Skeletal muscles from the *Lamp2*^y/−^ rats displayed substantial skeletal muscle fibre disarray and interstitial fibrosis (Fig. 4a). Additionally, PAS staining showed glycogen deposition in skeletal muscle fibres (Fig. 4b).

### Other system dysfunction of *Lamp2*-deficient rats

**Figure 5:**
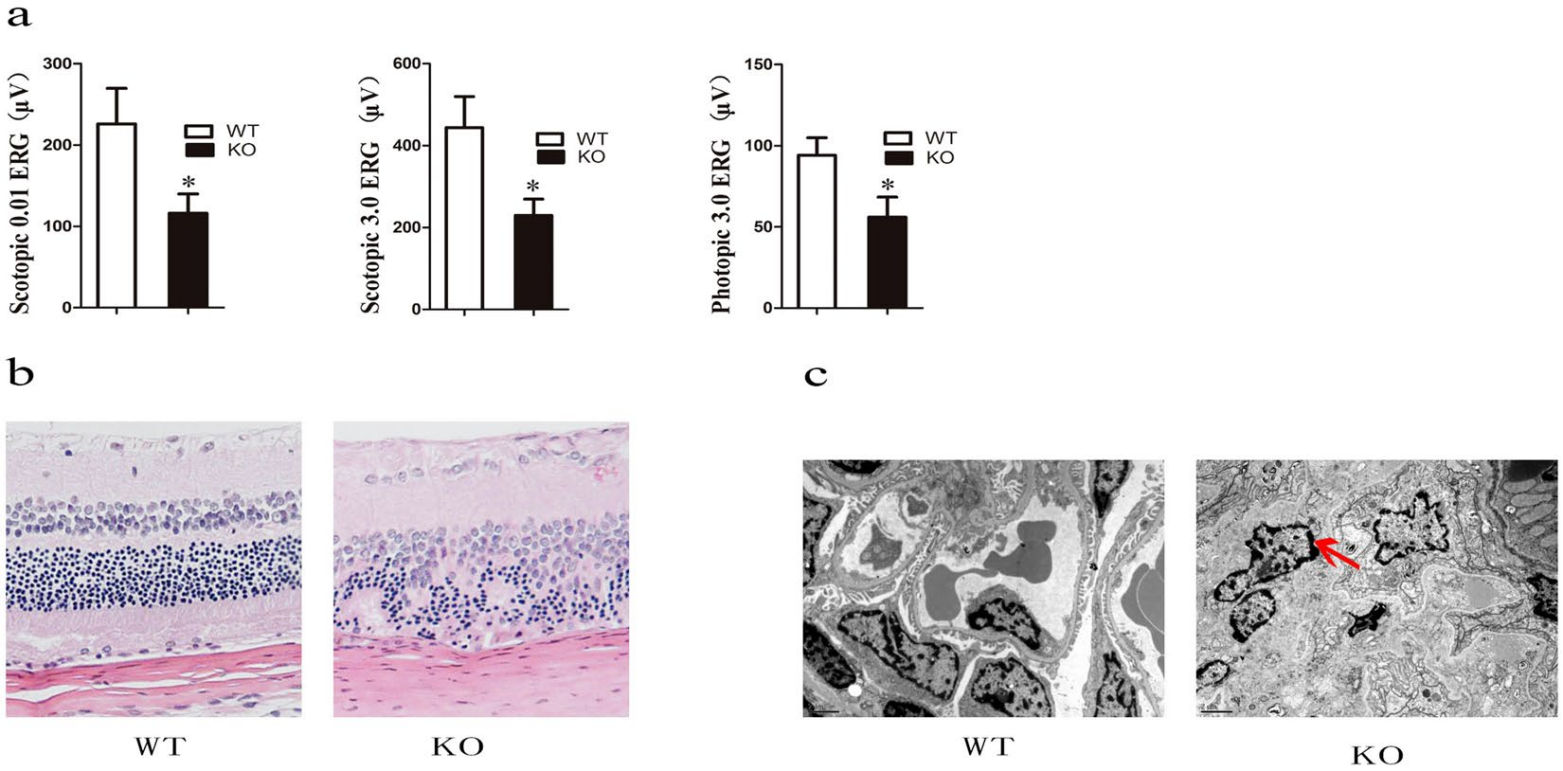
Changes in eyes and kidneys of *Lamp2*^y/−^ rats. (**a**) ERG analysis; (**b**) Paraffin sections with HE staining of retina; (**c**) Ultrastructure of the kidney. WT, wild-type male rats; KO, *Lamp2*^y/−^ rats.

In addition to the classic manifestations mentioned above, *Lamp2*^y/−^ rats were observed to display other system dysfunction. The eyes had chronic conjunctivitis with bleeding and tearing (Fig. S3a), and a number turned permanently pink or white. Electroretinogram (ERG) showed remarkably reduced β-wave amplitudes in scotopic 0.01 ERG, scotopic 3.0 ERG and photopic 3.0 ERG, indicating retinal function damage (Fig. 5a). HE staining showed that although no obvious abnormity in the retinal pigment epithelium was found, there was apparent cell arrangement disorder in outer and inner nuclear layers and the outer plexiform layer pinch (Fig. 5b), which could contribute to the attenuation of retinal function. While the retinal lesions of DD have not been thoroughly characterized to date, our results suggested *Lamp2* could play a vital role.

In addition, although there were increased nitrogen (BU) and creatinine (CRE) levels in serum (Table 1), and HE staining alterations (Fig. S3b) were not observed, the ultrastructure of the kidney in *Lamp2*^y/−^ rats revealed foot process fusion of podocytes, indicating chronic kidney injury.

Notably, no available *Lamp2*^−/−^ offspring due to *Lamp2*^y/−^ sterility were obtained after monitoring for nearly 3 years, and the mean litter number reduced to 8 or 9 from *Lamp2*^+/−^ females compared with 12 or 13 in wild-type female rats (data not shown), indicating that lack of *Lamp*2 might attenuate reproductive capacity.

## Discussion

DD is an extremely rare disorder with multisystem phenotypic variability and considerable clinical complexity. Therefore, animal models play a vital role in understanding the pathogenetic characteristics and processes. In our experiments, we comprehensively investigated the phenotypes of *Lamp2*^y/−^ rats and contrasted them with currently available mouse models. They exhibited hypertrophic cardiomyopathy, skeletal myopathy, and abnormal cognitive behaviour, accompanied by intrahepatic cholestasis, chronic conjunctivitis, retinopathy, chronic kidney injury, sterility, hypercholesterolemia and hyperglycaemia. Of note, *Lamp2*^y/−^ rats manifested more similar characteristics in terms of onset and multisystem lesions with DD patients.

DD cardiomyopathy typically manifests a hypertrophic phenotype. It was reported that the largest heart by weight ever reported in the medical literature was from a 14-year-old boy with a ventricular septal thickness of 65 mm and a weight of 1,425 grams at autopsy^13^. We observed that *Lamp2*^y/−^ rats showed prominently thicker LVPWd and IVSd and reduced LV end systole dimension from postnatal 48 days, and most died before 80 days. However, the rats’ LVEF was not affected, even though GCS and GRS were significantly reduced. These features of hypertrophic cardiomyopathy highly resembled those of DD male patients with preserved ejection fraction and normal cavity dimensions early in the course of the disease and only 11–12% dilated cavity later in progression to cardiac transplantation or death^2^. Although DD is an X-linked disease, and heterozygous female carriers are also affected, the clinical features of males and females are incomparable due to the different ages at presentation and clinical courses by gender. For example, female patients develop a more dilated cardiac phenotype and a progression of disease that occurs approximately 15 years later than in male counterparts^2,14^. As was reported previously, *Lamp2*-deficient heterozygous female mice primarily develop severely reduced cardiac contractility with a reduced ejection fraction and cardiac output, but no significant alteration of architecture index was observed at 30 weeks^6^. Few studies focus on the evaluation for hypertrophic cardiomyopathy of *Lamp2*-deficient hemizygote male mice. It has been proposed that transgenic mouse models are less suitable for serial non-invasive functional studies such as echocardiography or electrophysiology due to smaller cardiac size.

Although DD cardiomyopathy is a profound disease process characterized by rapid clinical deterioration leading to cardiac death in young patients <25 years, it is not suitable to ignore damage to other systems. Except for the classical traits of cardiomyopathy, skeletal myopathy and intellectual disability, DD patients may also suffer from other less prevalent symptoms, such as retinal disease (69% of males)^15–19^, hepatic disease^19–21^, respiratory disease (13/26, 50%), or gastrointestinal disease (20/26, 77%)^11^. Due to early death, birth and perinatal documents of DD patients are usually difficult to obtain. In our previous studies, *Lamp2*^y/−^ rats manifested the striking intrahepatic cholestasis with liver function abnormalities. Retinopathy with reduced ERG wave amplitudes, cell arrangement disorder in outer and inner nuclear layer and outer plexiform layer pinch were first reported in this research. Retinopathy was the first reported extra-cardiac manifestations in affected females, which could potentially be used to identify asymptomatic carriers^14^. Males affected with Danon disease demonstrated a near-complete loss of pigment in the retinal pigment epithelium^14^ and low electrooculogram voltages^18^. Of note, metabolic dysfunction with glycogen and unesterified cholesterol deposition has recently been reported in DD patients. We observed that *Lamp2*^y/−^ rats displayed not only the above described pathologic characteristics but also metabolic disorders, such as hypercholesterolemia and hyperglycaemia. Similarly, Lamp2-knockout mice displayed periodontitis because impaired neutrophils are unable to effectively eliminate bacterial pathogens^22^, and chronic conjunctivitis could be observed in *Lamp2*^y/−^ rats. In addition, chronic kidney injury and sterility were also found despite more details needing to be explored. Overall, *Lamp2*^y/−^ rats might represent a more severe pathology than that seen in DD patients in terms of the number of different organs affected. It has been proposed that defects in other tissues of individuals with DD might have been overlooked as the focus was on examining the severe cardiac dysfunction.

As an animal model, rats exhibited striking advantages over mice. The first obvious advantage was that the changes in heart structure were more notable earlier in rats than mice. Compared to wild-type male rats at 8 weeks, *Lamp2*^y/−^ rats manifested prominently thicker LVPWd, IVSd, and LVMI, but there was no significant difference in the left ventricular EF. However, LAMP-2 deficient mice displayed reduced EF and cardiac output, and there was no significant difference in LVPWd or LVMI compared to wild-type mice. We are tempted to speculate that the difference between rats and mice was primarily due to the different ages. The mean age of the mice was 30 weeks, and this old age may also have been responsible for the reduction in cardiac function. Rats in our study were from 4 weeks to 8 weeks old, and their cardiac structure had changed remarkably at 8 weeks, but the EF was in a range of control, probably because their cardiac function was still in a compensated stage. Second, rats showed multisystem damage, which was more similar to DD patients than mice. There was no report of other systems in mice, such as hepatic damage or retinopathy. Third, there were other advantages in rats. For instance, men are more susceptible to DD and exhibit more severe DD than females. The affected men have no offspring and usually die when they are adolescents. In fact, we observed that *Lamp2*^y/−^ rats mostly died after 60 days and were infertile, but *Lamp*2 deficient heterozygotes were mostly normal. However, it was reported that *Lamp*2 deficient homozygous mice could even living up to 19 months, and either homozygous or hemizygotes had normal fertility. In these terms, rats were closer to DD patients than mice. Additionally, *Lamp*2-deficient rats display more similar clinical features with DD patients compared to mouse models, and the physiological characteristics of rats are relatively closer to humans than mice. First, the heart rate of rats resembled that of humans, but mice had 5–10-fold higher heart rate than humans. Especially in mimicking some intricate morbid conditions, such as cardiovascular, metabolic, neurological and immune disorders, rats show definite advantages. In addition to the advantages mentioned above, the antidotal enzyme system in rats is also more similar to humans than to mice; therefore, rats are more accurate for detecting preclinical efficacy and toxicological testing of a new drug. Another advantage in rats is that their bodies are relatively larger than mice, which is more convenient for carrying out examinations, blood sampling, and surgical operations and for assess pharmacodynamics and drug safety. Therefore, transgenic rats should be widely used in medical research as a disease model.

Although *Lamp2*^y/−^ rats display many features similar to those seen in DD patients, many issues remain to be explored. First, there is a need to better establish and functionally characterize transgenic *Lamp*2 mutation or isoform-specific knockout animal models. Sixty-eight *Lamp*2 mutations have been reported so far^23^, most of which are splicing, large deletion, large duplication, insertion/deletion and missense mutations and could cause *Lamp*2 deficiency. There is evident genotype-phenotype correlation. For example, nonsense, frameshift and large deletion/duplication mutations showed the earliest age of onset^11^. In addition, *Lamp*2 is alternatively spliced to form three splice isoforms (*Lamp2A, Lamp2B*, *Lamp2C)*, whose different roles have been proposed in the autophagy process but their respective involvement in the DD phenotype seems unclear^24,25^. To date, only isoform-specific mutations have been reported for the *Lamp2B* isoform, and its deficiency seems to be a necessary and central feature in the pathogenesis of DD^2,23^. Second, mechanical or biochemical factors of *Lamp*2 deficiency associated phenotypes remain unknown despite autophagy being disrupted in the affected tissues. Fusion between the autophagosomes and lysosomes appears to be impaired^26^, whereas the relationship between *Lamp*2 and sarcomere proteins of myocyte thick or thin filament systems and mechanisms responsible formyocyte disarray are still undetermined. Our studies showed that *Lamp*2 could alter the location and expression of hepatocyte canalicular transporters, which contribute to liver damage^10^. Of note, *Lamp*2-deficient mice accumulate cholesterol in their liver and brain. This accumulation could be rescued by the lumenal domain and membrane-proximal part of *Lamp*2, suggesting that these regions of *Lamp*2 might have a crucial role in the transport of unesterified cholesterol^27^. In this study, glycogen accumulated within the affected model cells, both inside and outside of autophagic vacuoles^5^, which reflects the dysfunction of mitochondrial glycometabolism.

Taken together, the results of this study indicate that *Lamp*2^y/−^ rats are more similar to DD patients in severity, age of onset and clinical outcomes than are mouse models. Cardiomyopathy, skeletal-myopathy, and cognition symptoms were observed, as were several rare or novel phenotypes, such as intrahepatic cholestasis, chronic conjunctivitis, retinopathy, chronic kidney injury, sterility, hypercholesterolemia and hyperglycaemia. Therefore, this model might be used as a valuable animal model for DD.

## Methods

### Animals

Rats were fed in a 12-h light/dark cycle room and provided diet and water. All animal experimental protocols were approved by the Animal Welfare and Ethics Committee of the Fourth Military Medical University (FMMU), and carried out according to criteria outlined in Guide for the Care and Use of Laboratory Animals from the National Academy of Sciences (National Institutes of Health publication 86–23, 1985 revision).

### Echocardiography

All rats were carefully anaesthetized with 2% isoflurane and fixed on a heating pad (37 °C) in a supine position. Cardiac function was assessed across the thoracic region using 2-D guided M-mode echocardiography (Visual Sonics VeVo 770, Toronto, Canada) equipped with a 15–6 MHz linear transducer as previously described^28^. Left ventricular end-diastolic diameter (LVEDD), posterior wall thickness in diastole (LVPWd), interventricular septum thickness in diastole (IVSd), left ventricular mass index (LVMI) and ejection fraction (LVEF) were recorded. LVEF was calculated from end-diastolic volume (EDV) and end-systolic volume (ESV) using the equation of (EDV-ESV)/EDV. Estimated echocardiographically derived LV mass was calculated as [(LVEDD + septal wall thickness + posterior wall thickness) 3 − LVEDD3] × 1.055, where 1.055 (mg/mm^3^) denotes the density of the myocardium. LVMI was calculated as LV mass/body surface. Moreover, the echocardiographic images on the parasternal short-axis views were analysed offline using two-dimensional speckle tracking echocardiography. The global circumferential strain (GCS) and radial strain (GRS) of the left ventricle were assessed.

### Masson and PAS histological examination

Following anaesthesia, hearts and skeletal muscles were excised and immediately placed in 10% neutral-buffered formalin at room temperature for 24 h after a brief rinse with PBS. The specimens were embedded in paraffin and sliced into 4 μm thick sections for Masson and PAS staining. The representative areas were obtained using an Olympus microscope.

### Immunochemistry staining (MAb B7)

Formalin-fixed, paraffin-embedded sections were deparaffinized with xylene and rehydrated in graded ethanol (100 to 85%). After antigen retrieval, the endogenous peroxidase activity was blocked by 1% H_2_O_2_ for 30 min. Then, sections were washed and blocked with normal non-immunone goat serum for 30 min. Then, a hybridoma cell supernatant was applied on sections and incubated overnight at 4 °C. After washing with PBS, the sections were incubated 30 mins at room temperature with secondary antibody and then visualized with diaminobenzidine (Dako, Denmark).

### Open field test

The open field test was used to measure the spontaneous locomotor activity of the animals as previously described^29^. In detail, the apparatus was composed of four black acrylic plastic boxes (47 × 47 × 47 cm) (DigBehav, Jiliang Co., Ltd., Shanghai, China) placed in a soundproof box. Recordings were performed in the soundproof box illuminated by a red light (30 W). Rats were placed individually in the centre zone at the beginning of testing and allowed to freely explore the arena for 10 min. The total distance and locomotor activity in the entire field were recorded by an automatic analysis system. Subsequently, the distance, time and spontaneous activities in the central area (defined as the central 23.5 *23.5 cm^2^) were analysed. The apparatus was cleaned with a solution of 10% ethanol between trials to eliminate animal clues.

### Fear conditioning test

Experiments were performed as previously described^30^ in two contexts: (i) the shock chamber and (ii) the neutral test context. For shock application, rats were placed into the shock chamber (the chamber was cleaned with 10% ethanol), and after 196 s, a single 4 s scrambled electric shock of 1 mA current was applied to the feet via the metal grid and induced signs of pain (jumping or vocalizing). Stressed rats remained in the shock chamber for another 60 s before being returned to the home cages. To study cued memory, rats were placed back into the fear conditioning chamber for 3 min without tone presentation or a further shock application, and then immediately returned to the home cages 4 h after conditioning. To test contextual memory, animals were placed into the neutral test chamber. After 3 min, a neutral tone (CSn, 80 dB, 9 kHz) was presented for 3 min. After tone presentation, they remained in the test chamber for another 60 s before being returned to the home cages 4 h after conditioning. The recorded activities of all rats were analysed using an automatic analysis system (Freezing Scan, Clever Sys Inc., Reston, VA, USA).

### Electroretinography (ERG)

ERG was performed according to the previously described methods^31^. Briefly, rats were weighed and adapted to the dark over 12 hours before recording, and then anaesthetized by an intraperitoneal injection of 1% sodium pentobarbital (0.3 ml/100 g, Sigma-Aldrich) and Sumianxin II (0.025 ml/kg, Jilin Shengda Animal Pharmaceutical Co., Ltd., Jilin, China) under dim red light conditions. After anaesthesia, rats were lightly fixed to the stage and pupils were dilated with compound tropicamide eye drops (tropicamide, 5 mg/ml; phenylephrine hydrochloride, 5 mg/ml). Then, full-field ERG was recorded using the RETI-port system (Roland Consult, Havel, Germany) with custom-made silver chloride electrodes. The active electrode was placed at the cornea, and stainless steel needle electrodes were placed in subcutaneous tissue between the eyes. The stimulus was a brief white flash (3.0 cd-s/m^2^) delivered via a Ganzfeld integrating sphere. Signals were amplified and filtered to a bandpass of 1–300 Hz. A 50-Hz notch filter was used to eliminate line noise. A total of 60 photopic responses and 10 scotopic responses were recorded and averaged for a- and b-wave analysis. Oscillatory potentials (OPs) were isolated from the averaged recording traces using a 75 to 300-Hz digital filter. The magnitude of OPs was determined as the total of the major amplitudes. The amplitude of the OPs was measured from the bottom of the trough preceding each OP peak to the top of that peak.

### Statistical analysis

Mean ± SEM are presented in the figures. Comparisons between the groups were performed by Student’s t-tests or Mann–Whitney U-tests for parametric or non-parametric data sets, respectively. Statistical significance was accepted at *P* < 0.05.

## Electronic supplementary material

Supplementary Dataset 1
