# Supplementary material for: Characterisation of Lamp2-deficient rats for potential new animal model of Danon disease
Source: Sci Rep. 2018 May 2;8:6932. doi: 10.1038/s41598-018-24351-w (PMC5932014; doi:10.1038/s41598-018-24351-w)
Supplement: Supplementary file 1 — Supplementary Dataset 1 [file 41598_2018_24351_MOESM1_ESM.pdf]

Characterisation of Lamp2-deficient rats for potential new animal model of Danon disease

Shuoyi Ma[1*], Miao Zhang[1*], Shuai Zhang[1*], Jing Wang[2], Xia Zhou[1], Guanya Guo[1], Lu Wang[1], Min Wang[1], Zhengwu Peng[3], Changcun Guo[1], Xiaohong Zheng[1], Xinmin Zhou[1a], Jingbo Wang[1b], and Ying Han[1c]

1 State Key Laboratory of Cancer Biology, National Clinical Research Centre for Digestive Diseases and Xijing Hospital of Digestive Diseases, Fourth Military Medical University, Xi’an, China.

2 Division of Ultrasonography, Xijing Hospital, Fourth Military Medical University, Xi’an, China.

3 Division of Psychiatry, Xijing Hospital, Fourth Military Medical University, Xi’an, China.

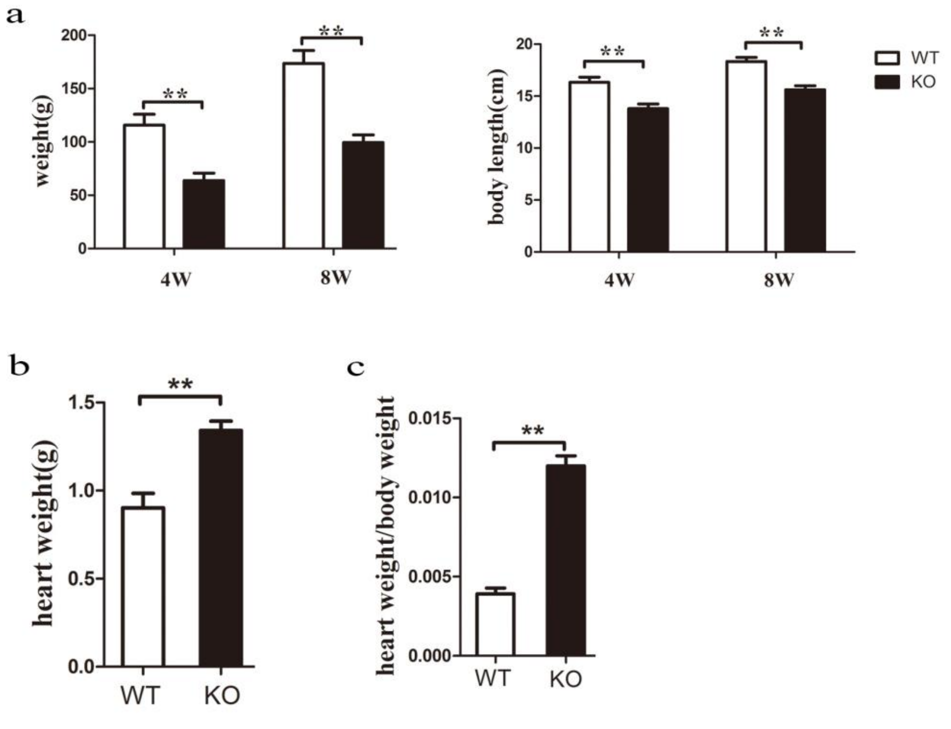

Supplementary Figure-1 (Han)

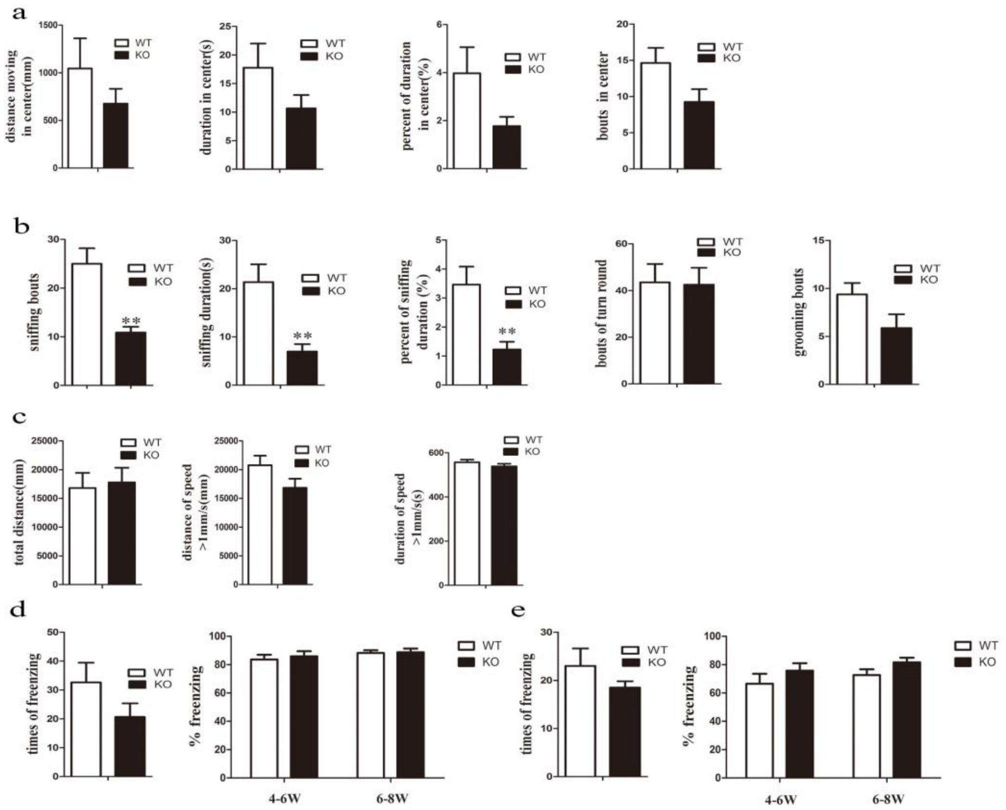

Supplementary Figure-2 (Han)

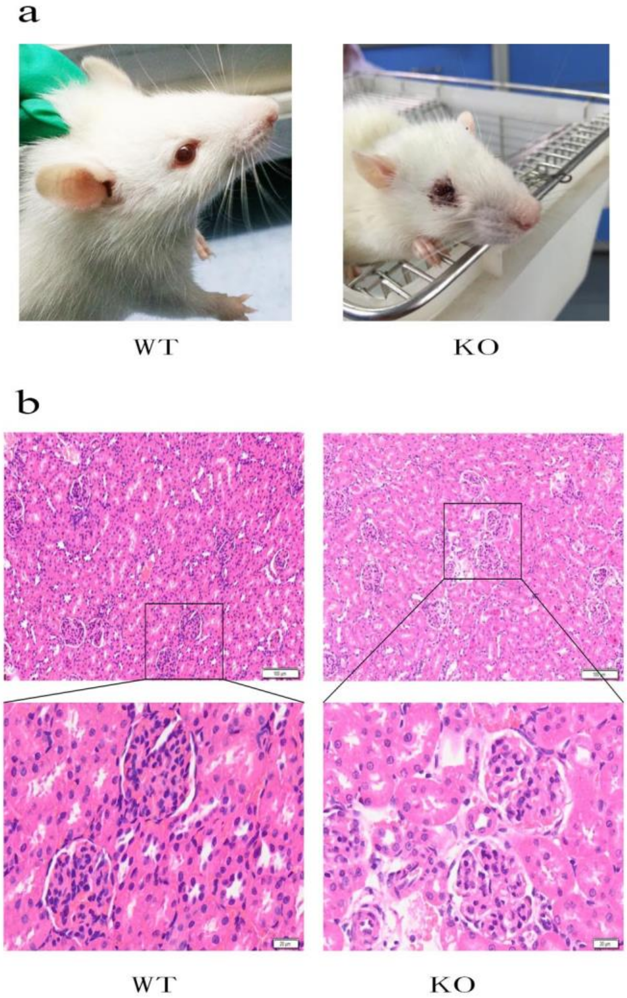

Supplementary Figure-3 (Han)
